# Ultrasound-based radiomics analysis for preoperative prediction of central and lateral cervical lymph node metastasis in papillary thyroid carcinoma: a multi-institutional study

**DOI:** 10.1186/s12880-022-00809-2

**Published:** 2022-05-02

**Authors:** Yuyang Tong, Jingwen Zhang, Yi Wei, Jinhua Yu, Weiwei Zhan, Hansheng Xia, Shichong Zhou, Yuanyuan Wang, Cai Chang

**Affiliations:** 1 Department of Ultrasound, Fudan University Shanghai Cancer Center, Department of Oncology, Shanghai Medical College, Fudan University, 270 Dong’an Road, Shanghai, 200032 China; 2 Department of Ultrasound, Ruijin Hospital, School of Medicine, Shanghai Jiao Tong University, Shanghai, 200025 China; 3 Department of Electronic Engineering, Fudan University and Key Laboratory of Medical Imaging Computing and Computer Assisted Intervention of Shanghai, Shanghai, 200433 China; 4 Department of Ultrasound, Zhongshan Hospital, Fudan University, 180 Fenglin Road, Shanghai, 200032 China

**Keywords:** Papillary thyroid carcinoma, Ultrasound, Radiomics, Nomogram, Cervical lymph node metastasis

## Abstract

**Background:**

An accurate preoperative assessment of cervical lymph node metastasis (LNM) is important for choosing an optimal therapeutic strategy for papillary thyroid carcinoma (PTC) patients. This study aimed to develop and validate two ultrasound (US) nomograms for the individual prediction of central and lateral compartment LNM in patients with PTC.

**Methods:**

A total of 720 PTC patients from 3 institutions were enrolled in this study. They were categorized into a primary cohort, an internal validation, and two external validation cohorts. Radiomics features were extracted from conventional US images. LASSO regression was used to select optimized features to construct the radiomics signature. Two nomograms integrating independent clinical variables and radiomics signature were established with multivariate logistic regression. The performance of the nomograms was assessed with regard to discrimination, calibration, and clinical usefulness.

**Results:**

The radiomics scores were significantly higher in patients with central/lateral LNM. A radiomics nomogram indicated good discrimination for central compartment LNM, with an area under the curve (AUC) of 0.875 in the training set, the corresponding value in the validation sets were 0.856, 0.870 and 0.870, respectively. Another nomogram for predicting lateral LNM also demonstrated good performance with an AUC of 0.938 and 0.905 in the training and internal validation cohorts, respectively. The AUC for the two external validation cohorts were 0.881 and 0.903, respectively. The clinical utility of the nomograms was confirmed by the decision curve analysis.

**Conclusion:**

The nomograms proposed here have favorable performance for preoperatively predicting cervical LNM, hold promise for optimizing the personalized treatment, and might greatly facilitate the decision-making in clinical practice.

**Supplementary Information:**

The online version contains supplementary material available at 10.1186/s12880-022-00809-2.

## Introduction

Papillary thyroid carcinoma (PTC) accounts for approximately 85% of all thyroid carcinoma cases, and its global incidence has been increasing over the past few decades [1]. Despite having an indolent clinical course, PTC is associated with cervical lymph node metastasis (LNM) [2]. Cervical LNM occurs in approximately 30–80% of PTC cases and is one of the most critical risk factors for locoregional recurrence [3]. The presence of LNM also affects the staging and treatment options for PTC [4]. Central compartment LNM is usually treated with central compartment neck dissection (CCND), and central LNM has been found in 45% of PTC patients with clinically negative LNs (cN0) who undergo prophylactic CCND [5]. However, the risk of hypoparathyroidism and recurrent laryngeal nerve injury is heightened with CCND; whether CCND should be performed prophylactically in cN0 patients remains controversial [6]. Patients with preoperatively evident lateral nodal metastasis typically require aggressive treatment, including lateral compartment lymph node (LN) dissection and high-dose radioactive iodine (RAI) therapy [7]. However, therapeutic neck dissection and/or RAI ablation increases the morbidity and reduces the quality of life [8]. Lowering the incidence of complication must be based on the reduction of unnecessary CLN dissection. Therefore, an accurate preoperative assessment of cervical LNM is important for choosing the optimal therapeutic strategy for PTC patients.

Ultrasound (US) is the preferred screening modality for preoperative assessment of cervical LN (CLN) status in PTC patients [9]. However, preoperative US has a low sensitivity (38–59%) for detecting cervical LNM [6, 10]. Furthermore, the diagnostic performance of neck US differs among physicians, and the interobserver variability is high [11]. Fine-needle aspiration (FNA) is an invasive approach, and its sensitivity for evaluating LNM varies and is specific to the operator [12]. Thus, a non-invasive, effective alternative imaging modality with improved accuracy is urgently needed in clinical practice.

Fueled by the rapid advances in medical imaging and developments in analytical methods, the field of radiomics has attracted increasing research attention in recent years [13]. Radiomics refers to the high-throughput extraction of extensive quantitative features to convert medical images into mineable data that could likely be used as diagnostic, predictive or prognostic biomarkers and support the clinical decision-making system [14]. Accordingly, our previous studies have proved the value of radiomics in predicting the cervical LNM of PTC [15–17]. To our knowledge, the US radiomics method applied in individually predicting cervical central and lateral LNM for PTC patients, respectively, has not been reported in the same article.

Therefore, the current study aimed to develop and validate nomograms that incorporated the US radiomics as well as the clinical risk factors for individual prediction of central and lateral cervical LNM in PTC patients.

## Materials and methods

### Patients

Between January 2019 to June 2019, consecutive patients with thyroid nodules in Fudan University Shanghai Cancer Center (Shanghai, China; training and internal validation cohort), Zhongshan Hospital (Shanghai, China; external validation cohort 1) and Ruijin Hospital (Shanghai, China; external validation cohort 2) were included. The inclusion criteria were as follows: (1) primary surgical resection was performed for the target tumor which had a pathological diagnosis of PTC; (2) preoperative neck US was performed, and the images were recorded and saved as DICOM format; (3) US images meet the requirements for the stored images [15]; (4) patients with complete clinicopathological information. The exclusion criteria included the following: (1) patients with more than one lesion confirmed to be PTC; (2) no history of preoperative therapy (radiofrequency or microwave ablation, neck radiation therapy); (3) patients with only microscopic cervical LNM. After exclusion, a total of 720 patients were enrolled in this study. Four hundred forty-three patients were chronologically divided into two cohorts: the training cohort with 300 patients who were treated between January and April 2019, and the internal validation cohort with 143 patients who were treated between May and June 2019. Two external validation cohorts enrolled 144 and 133 consecutive patients, respectively, using the same criteria. The patients were divided into two groups based on the pathological reports of the lymph node status after CLN dissection.

This retrospective study was approved by the Ethics Committees of all participating hospitals and performed in accordance with the Declaration of Helsinki. The inform consent was waived because of the retrospective nature of the study.

### Surgery and pathology

All patients received either total thyroidectomy or lobectomy in accordance with the clinical TNM staging and underwent therapeutic or prophylactic central compartment dissection. Lateral neck LN dissection was performed for patients with FNA proven, or radiologically suspicious LNs that were not suitable for FNA. The resected thyroid and LNs specimens were collected and subjected to pathological examination, including the determination of unifocal or multifocal, and tumor size (maximum diameter of the tumor); the number, size and neck level of the metastatic LNs. Experienced pathologists in each of the three institutions reviewed and validated the pathological results. The pathological results of the LNs were used as the gold standard [15].

### Clinical characteristics

The baseline clinical information, including age, gender, thyroglobulin (TG), thyroid stimulating hormone (TSH), thyroglobulin antibody (TGAB), thyroid peroxidase antibody (TPOAB), and cytological findings of FNA were collected from the medical record system and was double checked by the qualified specialists. The results of TG, TSH, TGAB, and TPOAB were detected within one week prior to surgical treatment. According to the previous reports and clinical experience [18, 19], the threshold set for TG, TSH, TGAB, and TPOAB were as follows: TG ≥ 77 ng/ml, TSH ≥ 2.5 ng/ml, TGAB ≥ 100 IU/ml, and TPOAB ≥ 35 IU/ml. Imaging data including US images, US reports and CT reports were obtained from the medical image station.

### US image acquisition and US-reported CLNs status

All patients received neck US examination before surgery. US images were acquired with a Supersonic Aixplorer System using a 15–4 linear-array transducer (SuperSonic Imaging, France) by radiologists with more than six years of experience. The US acquisition parameters were consistent among patients: image depth, 3 cm; focus parallel to the lesion, and gain 53%, and the spatial resolution of axial and lateral was 0.2 mm and 0.4 mm, respectively [20]. The detailed requirements for the US images were described in our previous study [20]. The diagnostic result “central LNM” or “lateral LNM” in the US report was defined as US-reported central/lateral cervical LNM positive. The results of “LNs negative”, “inflammatory LNs” and “small LNs” were categorized as US-reported central/lateral cervical LNM negative [15, 20]. The abnormal US finding suggestive of LNM include the following: (1) round shape; (2) absence of the fatty hilum; (3) microcalcification within the nodes; (4) presence of peripheral flow; (5) heterogeneity with cystic components [21]. LNs that met two or more of the five criteria would be considered positive. All the US images and reports were retrospectively reviewed and validated by independent senior radiologists. The diagnostic sensitivity, specificity and accuracy of neck US examination in the training and validation sets were calculated based upon the US reports [15].

### Region of interest (ROI) segmentation and feature extraction

A representative US image was selected, in which the tumor was delineated as the ROI, and was manually segmented by two senior US physicians with more than 6-year-experience in thyroid US imaging who were blinded from the final LNs status. Before feature extraction, the area inside the ROI where the feature extracted was min–max normalized into 0–255 to remove bias, and scaling factors of the effect of different imaging parameters. To avoid the effect of outliers, we calculated the maximum and minimum values after removing outliers for min–max normalization of images. Then, the software “PTC cervical LN metastasis prediction system” developed by the Department of Electronic Engineering, Fudan University [20] was used to input DICOM images after delineation, followed by extraction of image features. (MATLAB R2015b, Mathworks). To evaluate the reproducibility of feature extraction, fifty cases were randomly selected and double-blinded for comparing manual segmentation by two physicians. The interclass correlation coefficient (ICC) was calculated to measure the intra-observer and inter-observer agreement of the radiomics feature extraction. An ICC greater than 0.80 was considered to indicate good agreement.

### Feature selection and radiomics signature building

The detailed procedures of the feature selection and the radiomics signature construction for predicting the central and lateral cervical LNM were described in our previous studies [15, 16, 20], respectively. Briefly, based on the training set, 614 extracted reproducible features were reduced to 19 and 16 features, respectively, using our feature selection model. The selected radiomics features were then combined to build two radiomics signatures (Rad-score). Based on the estimated coefficient, a Rad-score for each case was computed to reflect the risk of cervical central and lateral LNM.

### Development of US radiomics nomogram

A multivariate logistic regression analysis comprising the Rad-score and the independent risk factors was conducted. The backward stepwise selection procedure was utilized with a liberal *p* value less than 0.05 as the retention criteria to select the final independent predictors for central/lateral LNM. Then a radiomics nomogram was developed based on the multivariate analysis in the training set.

### Performance of the US radiomics nomogram

The receiver operating characteristic curve (ROC) of the US-reported LN status, radiomics signature, and radiomics nomogram in each cohort was plotted. The discriminatory performance of the three models was evaluated using the area under curve (AUC). Calibration of the US radiomics nomogram in all four cohorts was assessed by the calibration curve and Hosmer–Lemeshow test. To determine the clinical usefulness of the US radiomics nomogram, decision curve analysis (DCA) was performed by quantifying the net benefits at different threshold probabilities in our entire dataset.

### Statistical analysis

The statistical analyses were performed with R software (version 3.5.3 http://www.r-project.prg), where the package ‘shiny’, ‘foreign’, ‘nomogramFormula’, ‘tmcn’, ‘rms’, ‘set’, ‘glmnet’, ‘rmda’ and ‘DT’ were applied. All other statistical tests were conducted using SPSS software (version 26, Chicago, IL). The categorical and normally distributed continuous variables were presented as frequency (percentage) and mean ± standard deviation (SD), respectively. Categorical variables were compared by *χ*^2^ test; student’s t-test was used for comparison between normally distributed continuous variables. Rad-scores were presented median (interquartile range), and the potential association of the Rad-scores and LN status in the training and validation cohorts were assessed using a Mann–Whitney U test. The Delong test was employed to compare different AUCs. A two-sided *p* < 0.05 was considered to indicate statistically significant.

## Results

### Clinical characteristics

The clinicopathological characteristics of the patients in the entire dataset are presented in Tables 1 and 2. The positive rate in terms of the central LNM in the training and validation cohorts were 34.7%, 32.9%, 48.6%, and 31.6%, respectively; and 9.0%, 10.5%, 10.4%, and 14.3%, respectively, with regard to the lateral LNM. No significant differences in the positive rate were found among the four cohorts (*p* = 0.058; *p* = 0.435).

**Table 1:** Associations between the central CLN status and clinical parameters in the training and validation cohorts

Characteristic	Training cohort (n = 300)			Validation cohort 1 (n = 143)		
	CLNM + (n = 104)	CLNM—(n = 196)	*p*	CLNM + (n = 47)	CLNM—(n = 96)	*p*
Age (years)			** < 0.001**			**0.038**
Mean ± SD	38.9 ± 10.6	45.3 ± 11.4		39.0 ± 11.4	43.6 ± 12.6	
Age (years)			** < 0.001**			**0.011**
> 45	28 (26.9)	94 (48.0)		13 (27.7)	48 (50.0)	
≤ 45	76 (73.1)	102 (52.0)		34 (72.3)	48 (50.0)	
Gender (%)			0.623			0.157
Male	32 (30.8)	55 (28.1)		16 (34.0)	22 (22.9)	
Female	72 (69.2)	141 (71.9)		31 (66.0)	74 (77.1)	
TSH (%)			0.487			0.975
Normal	93 (89.4)	180 (91.8)		44 (93.6)	90 (93.8)	
Abnormal	11 (10.6)	16 (8.2)		3 (6.4)	6 (6.3)	
TG (%)			0.143			0.482
Normal	92 (88.5)	183 (93.4)		45 (95.7)	89 (92.7)	
Abnormal	12 (11.5)	13 (6.6)		2 (4.3)	7 (7.2)	
TGAB (%)			0.518			0.111
Normal	64 (61.5)	128 (65.3)		27 (57.4)	68 (70.8)	
Abnormal	40 (38.5)	68 (34.7)		20 (42.6)	28 (29.2)	
TPOAB (%)			0.091			0.102
Normal	69 (66.3)	148 (75.5)		42 (89.4)	75 (78.1)	
Abnormal	35 (33.7)	48 (24.5)		5 (10.6)	21 (21.9)	
Tumor size (mm)			** < 0.001**			** < 0.001**
Mean ± SD	13.4 ± 8.1	9.2 ± 5.9		13.9 ± 10.1	9.0 ± 7.6	
Tumor size (mm)			** < 0.001**			** < 0.001**
≥ 1	64 (61.5)	53 (27.0)		29 (61.7)	15 (15.6)	
< 1	40 (38.5)	143 (73.0)		18 (38.3)	81 (84.4)	
US-reported central CLN status (%)			** < 0.001**			** < 0.001**
Negative	52 (50.0)	177 (90.3)		24 (51.1)	87 (90.6)	
positive	52 (50.0)	19 (9.7)		23 (48.9)	9 (9.4)	
Radiomics score Median	− 0.2873	− 0.4266	** < 0.001**	− 0.2631	− 0.4063	** < 0.001**
(Interquartile range)	(− 0.4481, − 0.1983)	(− 0.5547, − 0.3014)		(− 0.4026, − 0.1533)	(− 0.5130, − 0.2765)	

Characteristic	Validation cohort 2 (n = 144)			Validation cohort 3 (n = 133)		
	CLNM + (n = 70)	CLNM—(n = 74)	*p*	CLNM + (n = 42)	CLNM—(n = 91)	*p*
Age (years)			**0.017**			**0.014**
Mean ± SD	37.3 ± 10.9	41.9 ± 11.9		36.7 ± 12.1	45.2 ± 11.8	
Age (years)			0.052			**0.018**
> 45	22 (31.4)	35 (47.3)		12 (28.6)	46 (50.5)	
≤ 45	48 (68.6)	39 (52.7)		30 (71.4)	45 (49.5)	
Gender (%)			0.336			0.897
Male	20 (28.6)	16 (21.6)		12 (28.6)	27 (29.7)	
Female	50 (71.4)	58 (78.4)		30 (71.4)	64 (70.3)	
TSH (%)			0.920			0.551
Normal	64 (91.4)	68 (91.9)		38 (90.5)	85 (93.4)	
Abnormal	6 (8.6)	6 (8.1)		4 (9.5)	6 (6.6)	
TG (%)			0.095			0.335
Normal	44 (62.9)	56 (75.7)		27 (64.3)	66 (72.5)	
Abnormal	26 (37.1)	18 (24.3)		15 (35.7)	25 (27.5)	
TGAB (%)			0.143			0.596
Normal	40 (57.1)	51 (68.9)		32 (76.2)	73 (80.2)	
Abnormal	30 (42.9)	23 (31.1)		10 (23.8)	18 (19.8)	
TPOAB (%)			0.341			0.863
Normal	48 (68.6)	56 (75.7)		36 (85.7)	79 (86.8)	
Abnormal	22 (31.4)	18 (24.3)		6 (14.3)	12 (13.2)	
Tumor size (mm)			** < 0.001**			** < 0.001**
Mean ± SD	14.9 ± 7.1	8.3 ± 7.0		14.0 ± 11.2	8.3 ± 7.9	
Tumor size (mm)			** < 0.001**			**0.001**
≥ 1	41 (58.6)	20 (27.0)		33 (78.6)	20 (22.0)	
< 1	29 (41.4)	54 (73.0)		9 (21.4)	71 (78.0)	
US-reported central CLN status (%)			** < 0.001**			** < 0.001**
Negative	38 (54.3)	68 (91.9)		20 (47.6)	78 (85.7)	
Positive	32 (45.7)	6 (8.1)		22 (52.4)	13 (14.3)	
Radiomics score			** < 0.001**			** < 0.001**
Median	− 0.2431	− 0.4058		− 0.2695	− 0.4438	
(Interquartile range)	(− 0.3553, − 0.1270)	(− 0.5119, − 0.3104)		(− 0.3652, − 0.1542)	(− 0.5602, − 0.3217)	

CLN, cervical lymph node; CLNM, cervical lymph metastasis; TG, thyroglobulin; TGAB, thyroglobulin antibody; TPOAB, thyroid peroxidase antibody; TSH, thyroid stimulating hormone; LNM, lymph node metastasis; SD, standard deviation; US, ultrasound. Significant differences are highlighted in boldface

**Table 2:** Associations between the lateral CLN status and clinical parameters in the training and validation cohorts

Characteristic	Training cohort (n = 300)			Validation cohort 1 (n = 143)		
	LLNM + (n = 27)	LLNM—(n = 273)	*p*	LLNM + (n = 15)	LLNM—(n = 128)	*p*
Age (years)			0.515			0.543
Mean ± SD	40.1 ± 10.9	42.6 ± 12.0		41.5 ± 16.9	43.5 ± 11.0	
Age (years)			0.221			0.440
> 45	8 (29.6)	114 (41.8)		5 (33.3)	56 (43.8)	
≤ 45	19 (70.4)	159 (58.2)		10 (66.7)	72 (56.2)	
Gender (%)			0.159			0.542
Male	11 (40.7)	76 (27.8)		3 (20.0)	35 (27.3)	
Female	16 (59.3)	197 (72.2)		12 (80.0)	93 (72.7)	
TSH (%)			0.762			0.950
Normal	25 (92.6)	248 (90.8)		14 (93.3)	120 (93.8)	
Abnormal	2 (7.4)	25 (9.2)		1 (6.7)	8 (6.3)	
TG (%)			**0.001**			0.054
Normal	20 (74.1)	255 (93.4)		12 (80.0)	122 (95.3)	
Abnormal	7 (25.9)	18 (6.6)		3 (20.0)	6 (4.7)	
TGAB (%)			0.338			0.984
Normal	15 (55.6)	177 (64.8)		10 (66.7)	85 (66.4)	
Abnormal	12 (44.4)	96 (35.2)		5 (33.3)	43 (33.6)	
TPOAB (%)			0.254			0.847
Normal	17 (63.0)	200 (73.3)		12 (80.0)	105 (82.0)	
Abnormal	10 (37.0)	73 (26.7)		3 (20.0)	23 (18.0)	
Tumor size (mm)			** < 0.001**			** < 0.001**
Mean ± SD	15.1 ± 6.9	9.0 ± 6.8		16.1 ± 11.7	8.8 ± 6.7	
Tumor size (mm)			** < 0.001**			** < 0.001**
≥ 1	23 (85.2)	94 (34.4)		11 (73.3)	33 (25.8)	
< 1	4 (14.8)	179 (65.6)		4 (26.7)	95 (74.2)	
US-reported lateral CLN status (%)			** < 0.001**			** < 0.001**
Negative	7 (25.9)	243 (89.0)		4 (26.7)	116 (90.6)	
Positive	20 (74.1)	30 (11.0)		11 (73.3)	12 (9.4)	
Radiomics score Mean	− 0.0318	− 0.1346	** < 0.001**	− 0.0590	− 0.1414	** < 0.001**
(Interquartile range)	(− 0.1413, − 0.0081)	(− 0.1706, − 0.1114)		(− 0.1357, − 0.0323)	(− 0.1973–0.0986)	

Characteristic	Validation cohort 2 (n = 144)			Validation cohort 3 (n = 133)		
	LLNM + (n = 15)	LLNM—(n = 129)	*p*	LLNM + (n = 19)	LLNM—(n = 114)	*p*
Age (years)			0.992			**0.007**
Mean ± SD	39.7 ± 14.1	39.7 ± 11.4		38.8 ± 10.7	44.3 ± 12.3	
Age (years)			0.553			0.521
> 45	7 (46.7)	50 (38.8)		7 (36.8)	51 (44.7)	
≤ 45	8 (53.3)	79 (61.2)		12 (63.2)	63 (55.3)	
Gender (%)			0.270			0.816
Male	2 (13.3)	34 (26.4)		6 (28.9)	33 (31.6)	
Female	13 (86.7)	95 (73.6)		13 (71.1)	81 (68.4)	
TSH (%)			0.459			0.154
Normal	13 (86.7)	119 (92.2)		16 (84.2)	107 (93.9)	
Abnormal	2 (13.3)	10 (7.8)		3 (15.8)	7 (6.1)	
TG (%)			0.730			0.487
Normal	11 (73.3)	89 (69.0)		12 (63.2)	81 (71.1)	
Abnormal	4 (26.7)	40 (31.0)		7 (36.8)	33 (28.9)	
TGAB (%)			0.161			0.224
Normal	7 (46.7)	84 (65.1)		17 (89.5)	88 (77.2)	
Abnormal	8 (53.3)	45 (34.9)		2 (10.5)	26 (22.8)	
TPOAB (%)			0.084			0.679
Normal	8 (53.3)	96 (74.4)		17 (89.5)	98 (86.0)	
Abnormal	7 (46.7)	33 (25.6)		2 (10.5)	16 (14.0)	
Tumor size (mm)			** < 0.001**			** < 0.001**
Mean ± SD	14.9 ± 7.1	8.3 ± 7.0		15.7 ± 7.2	8.5 ± 6.9	
Tumor size (mm)			**0.002**			**0.001**
≥ 1	12 (80.0)	49 (38.0)		14 (73.7)	39 (34.2)	
< 1	3 (20.0)	80 (62.0)		5 (26.3)	75 (65.8)	
US-reported lateral CLN status (%)			** < 0.001**			** < 0.001**
Negative	4 (26.7)	112 (86.8)		5 (26.3)	99 (86.8)	
Positive	11 (73.3)	17 (13.2)		14 (73.7)	15 (13.2)	
Radiomics score			** < 0.001**			** < 0.001**
Mean	− 0.0629	− 0.1501		− 0.0496	− 0.1345	
(Interquartile range)	(− 0.1347, − 0.0033)	(− 0.2014, − 0.1020)		(− 0.1300, − 0.0029)	(− 0.1798, − 0.1008)	

CLN, cervical lymph node; CLNM, cervical lymph metastasis; TG, thyroglobulin; TGAB, thyroglobulin antibody; TPOAB, thyroid peroxidase antibody; TSH, thyroid stimulating hormone; LNM, lymph node metastasis; SD, standard deviation; US, ultrasound. Significant differences are highlighted in boldface

### Establishment of US radiomics signatures

In total, 614 US radiomics features were extracted from the original thyroid US images. Among them, features with nonzero coefficients were selected using our feature selection model [20]. The flowchart of the feature selection is presented in Additional file 1: Fig. S1, and the selected features in the radiomics score calculation formula can be found in Additional file 1: Table S1 and S2. The distribution of the Rad-score for patients with and without central/lateral LNM in the training and validation cohorts were displayed in Tables 1 and 2.

### Radiomics signature discrimination

**Fig. 1:**
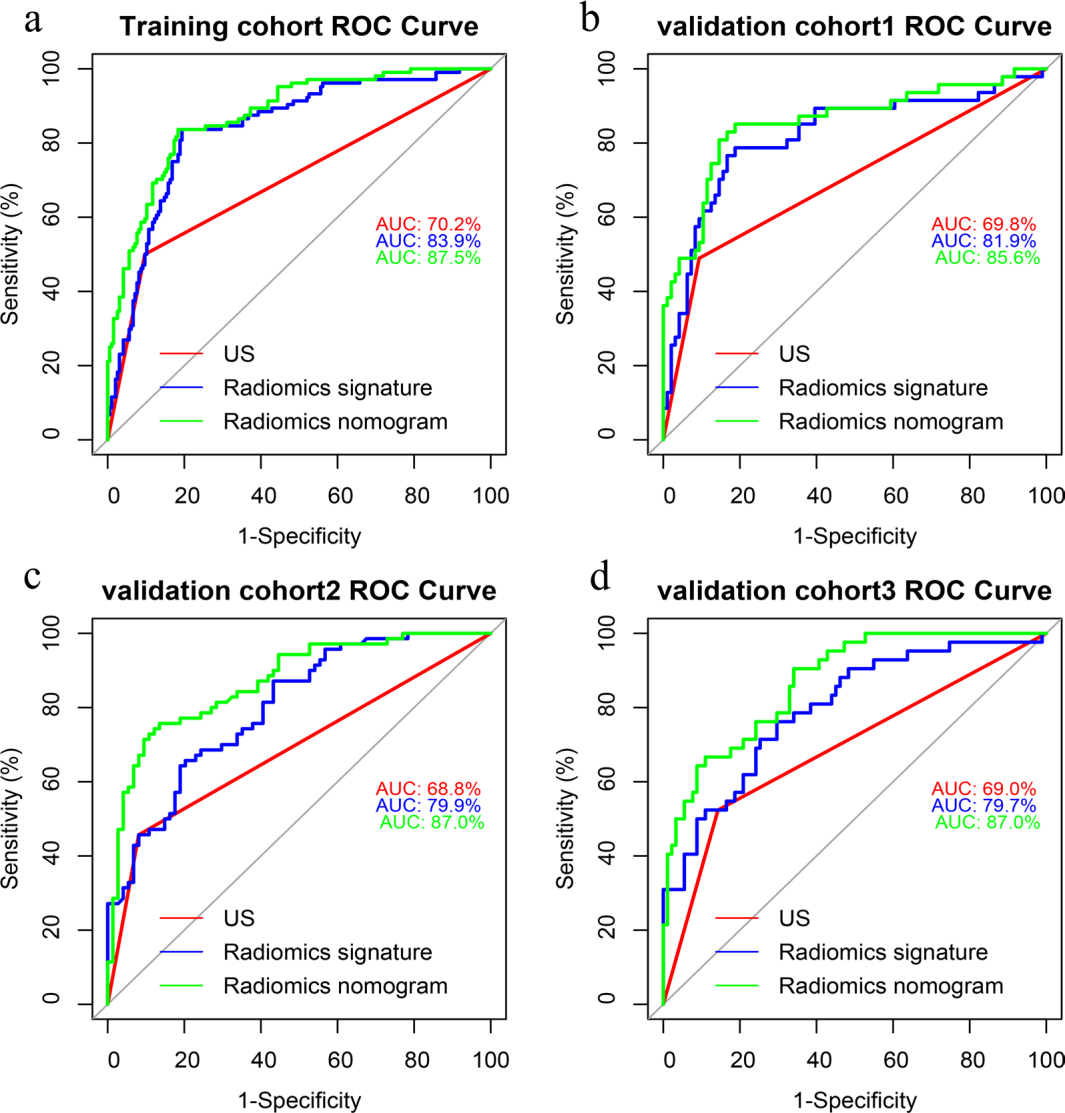
Performance of the different models in predicting the central LNM in PTC patients. **a** ROC curves of US-reported central LN status, radiomics signature, and radiomics nomogram for predicting central compartment LNM in the training cohort; **b** in the internal validation cohort; and **c**, **d** in two external validation cohorts. ROC, receiver operation characteristic; US, ultrasound; LN, lymph node; LNM, lymph node metastasis

**Fig. 2:**
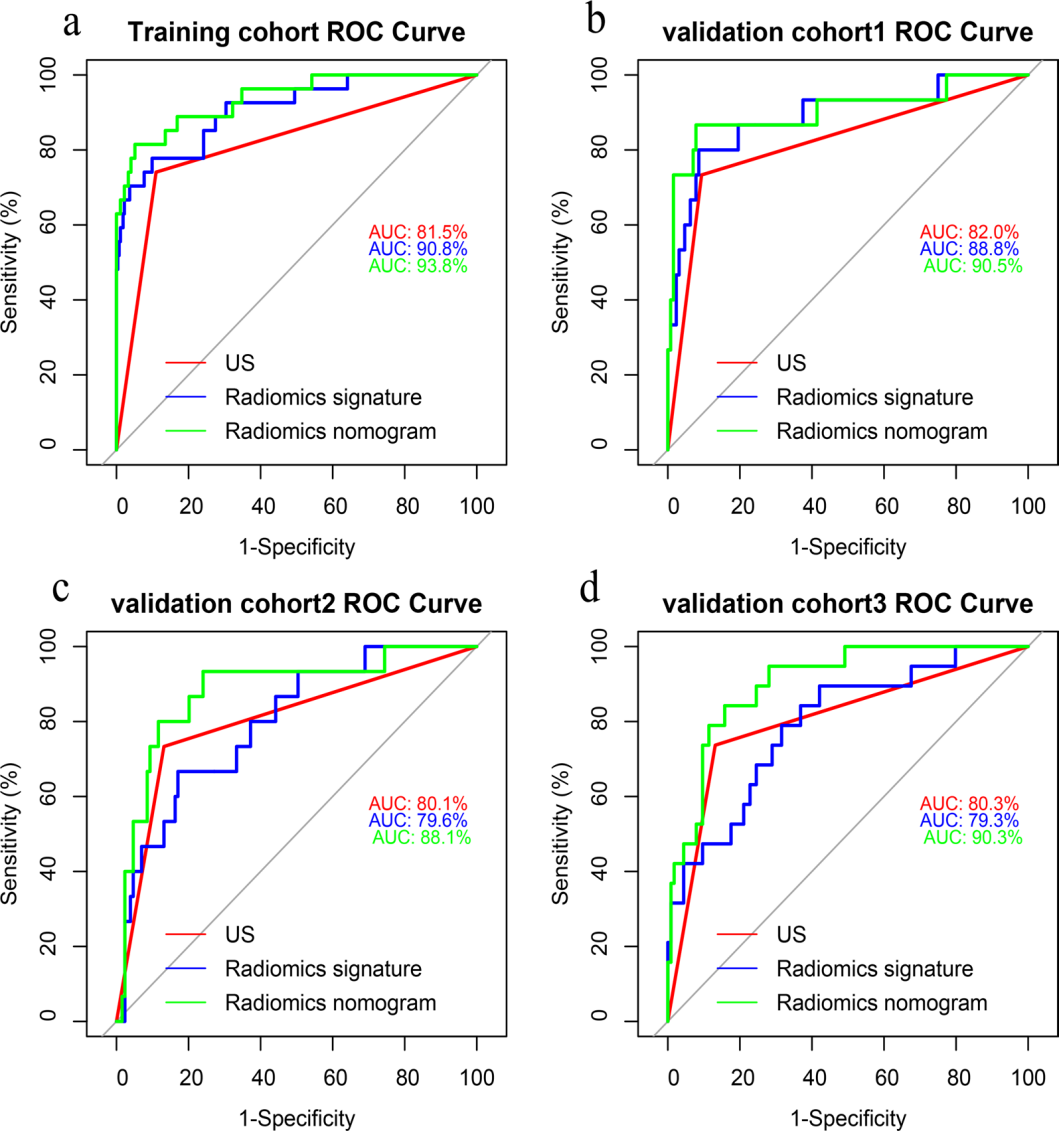
Performance of the different models in predicting the lateral LNM in PTC patients. **a** ROC curves of US-reported lateral LN status, radiomics signature, and radiomics nomogram for predicting lateral compartment LNM in the training cohort; **b** in the internal validation cohort; and **c**, **d** in two external validation cohorts. ROC, receiver operation characteristic; US, ultrasound; LN, lymph node; LNM, lymph node metastasis

The Rad-score was significantly higher in the central and lateral LNM groups than that in the non-LNM groups in both the training set and the validation sets (*p* < 0.001, *p* < 0.001, Tables 1 and 2). The radiomics signature yielded an AUC of 0.839 (cutoff value: − 0.389) for discriminating between central/non-central LNM groups in the training set, and 0.819, 0.799, 0.797, respectively, in three validation sets (Fig. 1). The AUC for differentiating between lateral/non-lateral LNM groups was 0.908 (cutoff value: -0.063) in the training set and were 0.888, 0.796, 0.793, respectively, in three validation cohorts (Fig. 2). The radiomics signatures yielded a better discriminatory performance of cervical LNM than that of the radiologists’ subjective prediction (Tables 3 and 4).

**Table 3:** Diagnostic performance of US, radiomics signature, and nomogram for predicting central LN status in the training and validation cohorts

Cohorts	Sensitivity (%)	Specificity (%)	AUC (95%CI)	Accuracy (%)
*Training cohort*				
US-reported central LN status in training cohort	50.0	90.3	0.702 (0.635–0.768)	76.3
Radiomics signature in training cohort	83.7	81.1	0.839 (0.792–0.887)	82.0
Radiomics nomogram in training cohort	83.7	81.6	0.875 (0.834–0.915)	82.3
*Internal validation cohort*				
US-reported central LN status in validation cohort 1	48.9	90.6	0.698 (0.599–0.797)	76.9
Radiomics signature in validation cohort 1	78.7	81.2	0.819 (0.738–0.900)	80.4
Radiomics nomogram in validation cohort 1	83.0	82.3	0.857 (0.785–0.929)	82.5
*External validation cohort 1*				
US-reported central LN status in validation cohort 2	45.7	91.9	0.688 (0.600–0.776)	69.4
Radiomics signature in validation cohort 2	65.7	79.7	0.799 (0.729–0.869)	72.9
Radiomics nomogram in validation cohort 2	77.1	85.1	0.880 (0.826–0.934)	81.3
*External validation cohort 2*				
US-reported central LN status in validation cohort 3	52.4	85.7	0.690 (0.587–0.794)	75.1
Radiomics signature in validation cohort 3	71.4	74.7	0.797 (0.716–0.879)	73.7
Radiomics nomogram in validation cohort 3	71.4	87.9	0.870 (0.808–0.932)	82.7

US, ultrasound; CT, computed tomography; AUC, area under curve; CI, confidence interval

**Table 4:** Diagnostic performance of US, radiomics signature, and nomogram for predicting lateral LN status in the training and validation cohorts

Cohorts	Sensitivity (%)	Specificity (%)	AUC (95%CI)	Accuracy (%)
*Training cohort*				
US-reported lateral LN status in training cohort	74.1	89.0	0.815 (0.716–0.915)	87.6
Radiomics signature in training cohort	77.8	90.1	0.908 (0.845–0.972)	89.0
Radiomics nomogram in training cohort	81.5	94.1	0.938 (0.887–0.989)	93.0
*Internal validation cohort*				
US-reported lateral LN status in validation cohort	73.3	90.6	0.820 (0.684–0.955)	88.8
Radiomics signature in validation cohort 1	80.0	91.4	0.888 (0.786–0.990)	90.2
Radiomics nomogram in validation cohort 1	86.7	92.2	0.905 (0.789–0.999)	91.6
*External validation cohort 1*				
US-reported lateral LN status in validation cohort 2	73.3	86.8	0.801 (0.665–0.936)	85.4
Radiomics signature in validation cohort 2	66.7	82.9	0.796 (0.684–0.909)	84.7
Radiomics nomogram in validation cohort 2	73.3	93.0	0.884 (0.785–0.984)	90.9
*External validation cohort 2*				
US-reported lateral LN status in validation cohort 3	73.7	86.8	0.803 (0.681–0.924)	85.0
Radiomics signature in validation cohort 3	68.4	75.4	0.793 (0.683–0.903)	74.4
Radiomics nomogram in validation cohort 3	78.9	89.5	0.906 (0.841–0.971)	88.0

US, ultrasound; CT, computed tomography; AUC, area under curve; CI, confidence interval

### Prediction of LN status based on radiomics nomogram

**Fig. 3:**
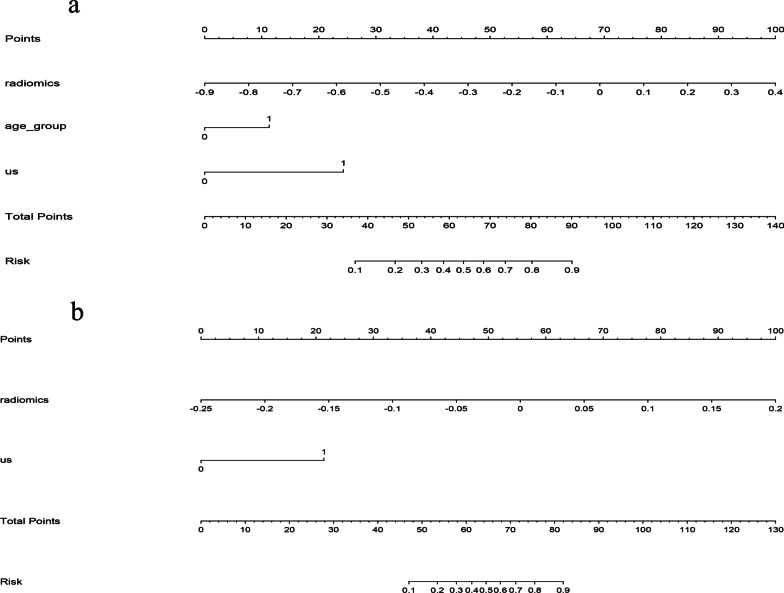
US-based radiomics nomograms used for prediction of **a** central compartment and **b** lateral compartment LNM in patients with PTC. US, ultrasound; LNM, lymph node metastasis; PTC, papillary thyroid carcinoma

**Fig. 4:**
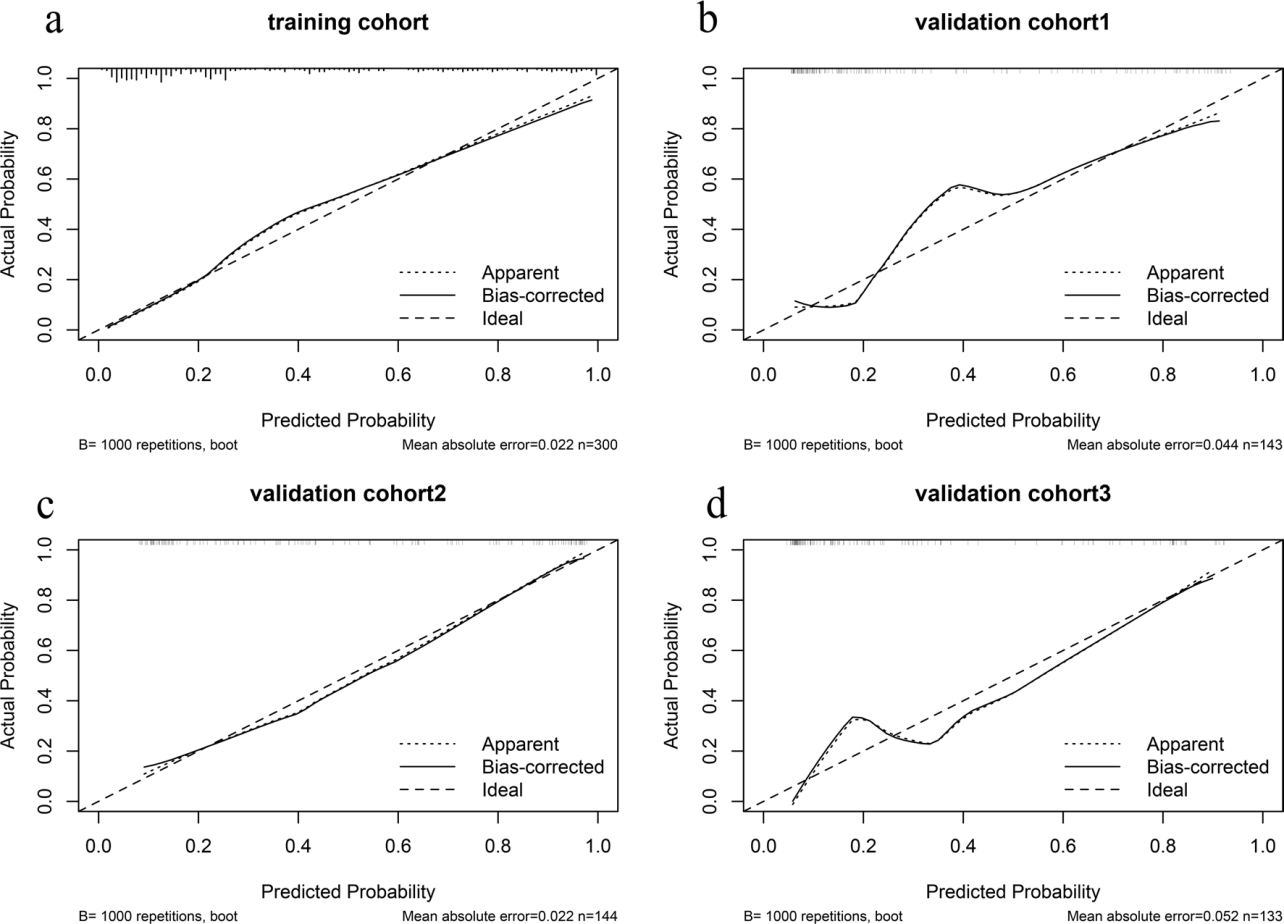
Calibration curve evaluate the radiomics nomogram used for prediction of central LNM **a** in the training cohort (*p* = 0.182), **b** internal cohort (0.427), and **c**, **d** in two external cohorts (*p* = 0.561, *p* = 0.894). The Hosmer–Lemeshow tests yield nonsignificant statistics. LNM, lymph node metastasis

**Fig. 5:**
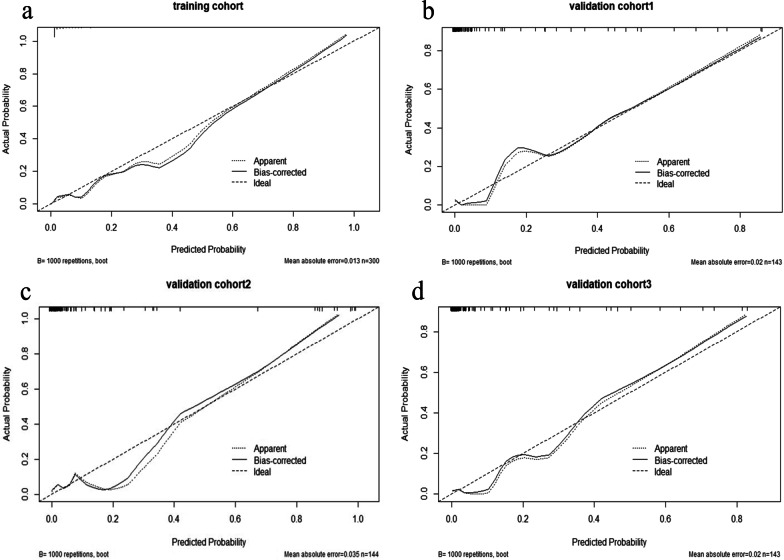
Calibration curve evaluate the radiomics nomogram used for prediction of lateral LNM **a** in the training cohort (*p* = 0.722), **b** internal cohort (*p* = 0.326), and **c**, **d** in two external cohorts (*p* = 0.637, *p* = 0.589). The Hosmer–Lemeshow tests yield nonsignificant statistics. LNM, lymph node metastasis

The age (< 45), US-reported central CLN status, and radiomics signature were identified as independent predictive factors for central LNM. The US-reported lateral CLN status and radiomics signature were identified as independent variables for lateral LNM by multivariate logistic regression analysis (Tables 5 and 6). The two radiomics nomograms were constructed by incorporating the independent predictors. In the training sets, the radiomics nomogram showed the highest discrimination between central LNM positive and negative with an AUC of 0.875 (95% CI 0.834–0.915; cutoff value: 0.273; ~ 52 points; Fig. 3a); the nomogram for discriminating between the lateral LNM positive and negative also yielded the greatest AUC (0.938, 95% CI 0.887–0.989; cutoff value: 0.177; ~ 52 points; Fig. 3b), which indicated that nomogram achieved better discriminatory performance than either the US-reported CLN status or the radiomics signature. The favorable discrimination was also observed in the validation cohorts (Tables 3 and 4). The calibration curve of the radiomics nomograms for the probability of central/lateral LNM demonstrated an optimal consistency between the prediction and pathologic observation in the training and validation cohorts (Figs. 4 and 5). The Hosmer–Lemeshow test showed no statistical significance, which suggested no significant deviation from a perfect fit.

**Table 5:** Independent predictive factors for central LNM in the radiomics nomogram

Variables	*β*	Odds ratio (95% CI)	*p*
Age	0.934	2.544 (1.317–4.913)	0.005
US-reported central CLN status	2.007	7.444 (3.597–15.406)	< 0.001
US radiomics signature	6.354	574.537 (96.796–3410.181)	< 0.001
Constant	0.473		0.225

*β* is the regression coefficient

CI, confidence interval; CLN indicates cervical lymph nodes; US, ultrasound

**Table 6:** Independent predictive factors for lateral LNM in the radiomics nomogram

Variables	*β*	Odds ratio (95% CI)	*p*
US-reported central CLN status	25.752	142.012 (28.751–701.016)	< 0.001
US radiomics signature	2.693	14.772 (4.312–50.607)	< 0.001
Constant	− 1.125		0.031

*β* is the regression coefficient

CI, confidence interval; CLN indicates cervical lymph nodes; US, ultrasound

### Clinical significance

**Fig. 6:**
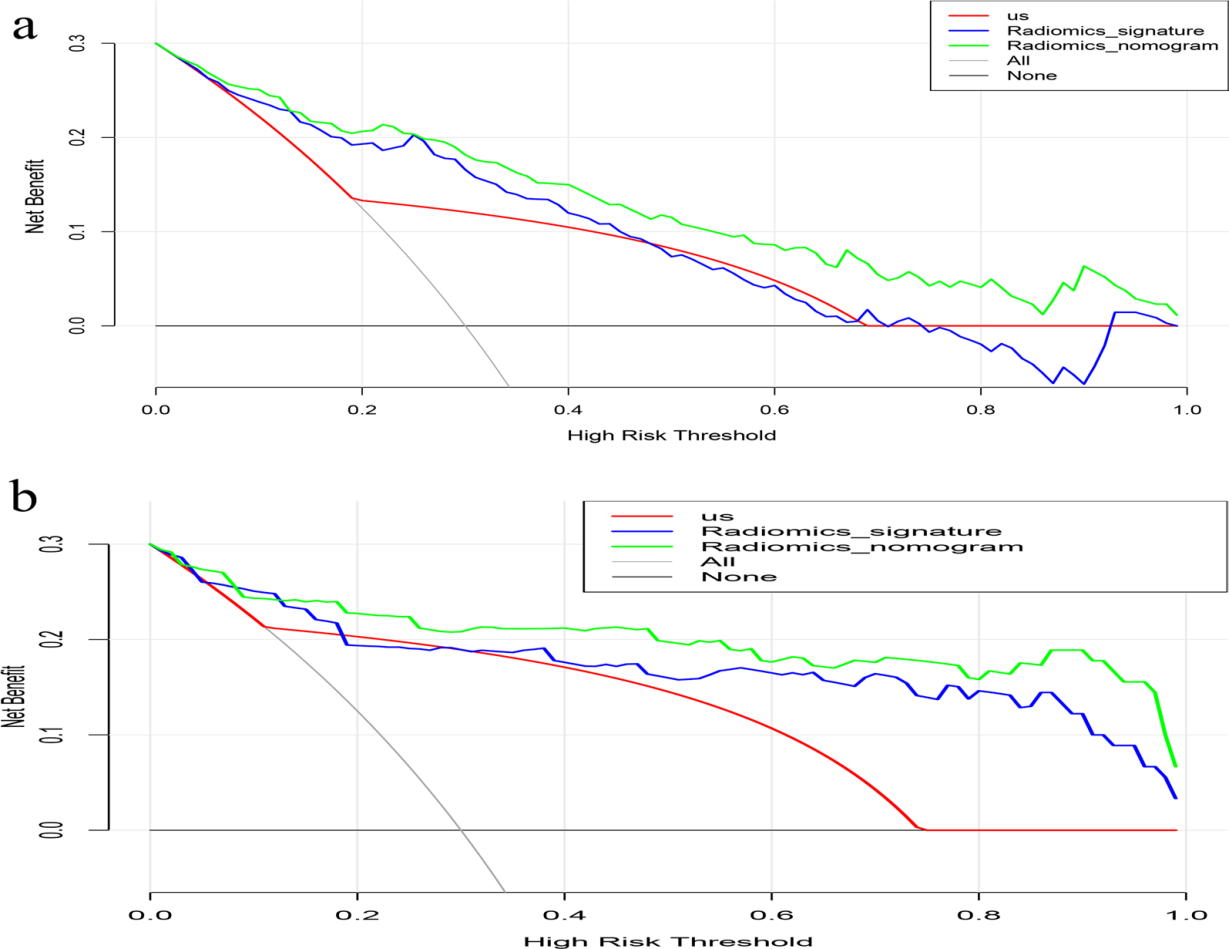
Decision curve analysis for each model in predicting **a** central compartment and **b** lateral compartment LNM in PTC patients. The y-axis represents the net benefit, which was calculated by summing the benefits (true positives) and subtracting the harms (false positives), and weighing the relative harm of false-positive and false-negative results. According to threshold probability obtained, the radiomics nomograms have the greatest net benefit compared with other models or simple strategies such as all-treat and non-treat scheme do. LNM, lymph node metastasis; PTC, papillary thyroid carcinoma

The decision curve analyses (DCA) for the US-reported CLN status, the radiomics signature, and the nomograms are illustrated in Fig. 6. The DCA demonstrated that, compared with other models, the nomogram exhibited an optimal net benefit to predict the central LNM for threshold probability within the range of 0–0.98. And the prediction of lateral LNM with radiomics nomogram could benefit more as compared to the radiomics signature and the US-reported lateral CLN status when the threshold probability ranged from 0.04 to 0.98.

## Discussion

In this multi-institutional study, we developed and validated two radiomics nomograms to preoperatively predict the central and lateral LNM, respectively, in a non-invasive and individualized fashion. These two nomograms showed favorable predictive ability in both training and independent validation cohorts, outperforming the radiologists’ subjective prediction of cervical LNM and the radiomics signature. Furthermore, the DCA also demonstrated that the nomograms had the best net benefit within a wide range of threshold probabilities. These results highlighted that our radiomics nomograms provided a new method for an individualized prediction of central and lateral LNM in PTC patients before surgery.

Accurate preoperative prediction of the cervical LNM of patients with PTC is of great importance for guiding clinical treatment, particularly for surgeons to determine the extent of the surgical resection and assess the necessity of CLN dissection [3, 13]. Neck US examination plays a pivotal role in PTC staging. Unfortunately, the detection rate of cervical LNM is not desirable. Since US is unable to consistently visualize the deep anatomic structures or structures that are acoustically shadowed by air and bone [22], in many patients, the LNM in the central compartment may not show any abnormal finding in preoperative US examination [23]. Besides, US examination is an empirical diagnosis which is greatly affected by the expertise of the operator; therefore, prone to interobserver variability when determining lateral LNM [8]. Many studies have investigated the association between cervical LNM and the morphologic US features of the primary tumor, and have reported that the “taller than wide” shape, tumor size, presence of calcification, and closer distance between tumor and capsule were independent risk factors for LNM [15, 24–26]. Although the US features mentioned above are encouraging, the imaging features are also based on the judgment of the performing physician and thus, lack objectivity. However, utilizing imaging features of the primary tumor enables an approach that is less affected by the expertise of the operator, thus, could be a promising tool for clinical practice [8].

Currently, radiomics has been explored in the field of thyroid carcinoma research, such as in the differential diagnosis of thyroid nodules [27], prediction of the cervical LNM [16], extrathyroidal extension [28], BRAF^V600E^ mutation [29], as well as the survival prediction [30]. Lu et al. developed a nomogram based on the contrast-enhanced CT, which showed favorable performance in predicting LNM in PTC patients with an AUC of 0.867 [31]. Nevertheless, iodinated contrast agents have a risk of contrast allergy and might delay the RAI in PTC patients, which limits its clinical application. Hu et al. established an MRI radiomics model, which obtained good accuracy in predicting LNM status preoperatively [6]. However, MRI is expensive, time-consuming, and not routinely performed for PTC patients in clinical management.

Our previous study has demonstrated the feasibility of applying the US-based radiomics analysis for assessing the risk of LNM in PTC patients [16]. Jiang et al. established a nomogram that combined the shear-wave elastography (SWE) radiomics signature and clinicopathological parameters achieved satisfactory predictive value for LN staging in PTC patients, with an AUC of 0.851 and 0.832 in the training and validation cohort, respectively [23]. These US radiomics studies were designed to predict the cervical LNM; however, the treatment strategy for LNM in the central or lateral compartment varies greatly. Thus, accurate radiomics models for predicting LNM in central or lateral compartments, respectively, are urgently needed. Then, another two US radiomics studies were conducted by us separately. One was designed to build a radiomics nomogram which could preoperatively predict the central LNM in PTC patients. The other was designed to develop a radiomics model that could discriminate the lateral LNM prior to surgery. Both of the studies achieved desirable results and was validated by a single independent validation set. However, the effectiveness of the two models needs to be validated in a larger multi-institutional study.

Here, we investigated the association between central/ lateral LNM and preoperatively available characteristics by univariate analysis. By multivariate logistic regression analyses, age, US-reported central CLN status, and radiomics signature were identified as the independent predictors for central LNM; US-reported lateral CLN status and radiomics signature were identified as the independent risk factors for lateral LNM. Then, two nomograms were developed based on the multivariate analyses. Interestingly, the independent risk factors on which the two nomograms were based in this study were also the independent risk factors for central/lateral LNM in our previous studies [15, 20]. This may indicate the great predictive value of these factors for cervical LNM in PTC patients. The nomograms have been confirmed to be capable of generating a probability of central/lateral LNM preoperatively and individually, which is in line with the prevailing concept of personalized precision medicine. The calibration curve showed superior consistency between nomogram-estimated and actual observed probability in each of the cohorts. For the clinical usefulness of the nomograms, we employed DCA to evaluate whether the nomogram-assisted decision-making would improve patient outcomes. Our results demonstrated that the nomogram supplied better clinical net benefit across the majority range of reasonable threshold probabilities than either the radiomics signature or the US-reported CLN status.

Some limitations have to be acknowledged in this study. First, the gene mutation status was not included in this study. Recently, increasing studies have been conducted to investigate the association between gene mutation and the LNM in PTC. Since not all the patients received the BRAF mutation examination after FNA, the role of gene mutation as an independent predictor still needs to be further studied. Second, the radiomics features were only extracted from conventional US images. Since multimodal US technology, such as SWE and the contrast-enhanced US, has been applied jointly for the diagnosis of thyroid nodules in clinical practice, a new radiomics study incorporating multimodal technology is ongoing at our center. Third, the utilized images were obtained from the same US system. Radiomics features have been reported to be affected by the US machine and parameters used for image acquisition. Thus, a multi-center study with multiple US systems is needed to acquire high-level evidence for further clinical application.

In conclusion, we developed an easy-to-use and noninvasive predictive tool that incorporates the radiomics signature and clinical characteristics to preoperatively evaluate the individual risk of cervical central/lateral LNM in patients with PTC. The nomograms proposed here hold promise for optimizing personalized treatment and might greatly facilitate the decision-making in clinical practice.

## Supplementary Information

**Additional file 1**. **Figure S1.** The flowchart of the radiomics feature selection. a) Procedure of features selection for establishing the predictive model for central LNM; b) procedure of features selection for developing the predictive model for lateral LNM. ICC, inter-and intra-class correlation coefficients; GA, genetic algorithm; mRMR, minimum redundancy maximum relevance; LASSO, least absolute shrinkage and selection operator; LNM, lymph node metastasis. **Table S1.** Radiomic features for predicting central LNM and weighting coefficients after LASSO regression. **Table S2.** Radiomic features for predicting lateral LNM and weighting coefficients after LASSO regression.

## Data Availability

The datasets used and analyzed during the current study are available from the corresponding author on reasonable request.

## Abbreviations

LNM: Lymph node metastasis
PTC: Papillary thyroid carcinoma
US: Ultrasound
CLN: Cervical lymph node
LN: Lymph node
CT: Computed tomography
FNA: Fine-needle aspiration
CCND: Central compartment neck dissection
RAI: Radioactive iodine
Rad-score: Radiomics score
AUC: Area under the ROC curve
TSH: Thyroid stimulating hormone
TG: Thyroglobulin
TPOAB: Thyroglobulin antibody
ICC: Interclass correlation coefficient
LASSO: Least absolute shrinkage and selection operator
ROC: Receiver operating characteristic
DCA: Decision curve analysis
SD: Standard deviation
SWE: Shear-wave elastography
