# Supplementary material for: Ultrasound-based radiomics analysis for preoperative prediction of central and lateral cervical lymph node metastasis in papillary thyroid carcinoma: a multi-institutional study
Source: BMC Med Imaging. 2022 May 2;22:82. doi: 10.1186/s12880-022-00809-2 (PMC9059387; doi:10.1186/s12880-022-00809-2)
Supplement: Supplementary file 1 — Additional file 1. Figure S1. The flowchart of the radiomics feature selection. a) Procedure of features selection for establishing the predictive model for central LNM; b) procedure of features selection for developing the predictive model for lateral LNM. ICC, inter-and intra-class correlation coefficients; GA, genetic algorithm; mRMR, minimum redundancy maximum relevance; LASSO, least absolute shrinkage and selection operator; LNM, lymph node metastasis. Table S1. Radiomic features for predicting central LNM and weighting coefficients after LASSO regression. Table S2. Radiomic features for predicting lateral LNM and weighting coefficients after LASSO regression. [file 12880_2022_809_MOESM1_ESM.docx]

**Ultrasound-based radiomics analysis for preoperative prediction of central and lateral cervical lymph node metastasis in papillary thyroid carcinoma: a multi-institutional study**

**Yuyang Tong****, Jingwen Zhang, Yi Wei, Jinhua Yu, Weiwei Zhan, Hansheng Xia, Shichong Zhou, Yuanyuan Wang, Cai Chang**

**Supplementary materials**

**Figure S1**


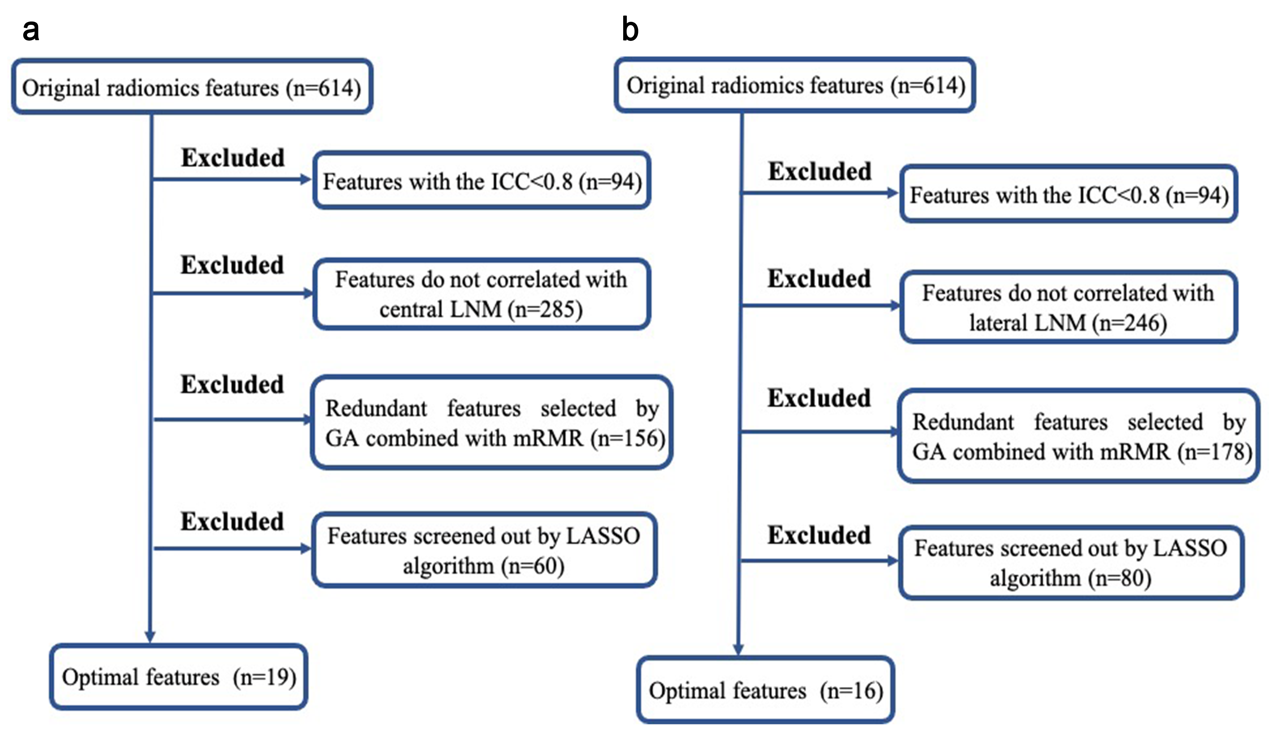


Figure S1: The flowchart of the radiomics feature selection. a) Procedure of features selection for establishing the predictive model for central LNM; b) procedure of features selection for developing the predictive model for lateral LNM. ICC, inter-and intra-class correlation coefficients; GA, genetic algorithm; mRMR, minimum redundancy maximum relevance; LASSO, least absolute shrinkage and selection operator; LNM, lymph node metastasis.

**Table S1.** Radiomic features for predicting central LNM and weighting coefficients after LASSO regression

| Lasso weighted coefficient | US radiomics feature in formula |
| --- | --- |
| −0.01156 | Cal Area c min |
| 0.13760 | Cal Area c max |
| 0.08604 | Cal SD of Roundness |
| 0.15802 | Cal sum of Perimeter |
| −0.02044 | M Con SD p |
| −0.02300 | M compactness |
| −0.12641 | M Orientation |
| 0.21770 | M spiculation-1 |
| 0.138438 | M spiculation-2 |
| −0.01144 | M overlap Area |
| -0.02450 | Histogram t MAD |
| 0.04142 | Histogram p entropy |
| 0.03154 | Histogram p Range |
| −0.15004 | GlszmTextures-3.Zone-Size Variance |
| −0.06091 | GlszmTextures-2.Zone-Size Variance |
| 0.03266 | Glszm.Gray-Level Nonuniformity |
| 0.07844 | Glszm.Low-Gray-Level Zone Emphasis |
| −0.05400 | Glcm.Maximum Probability |
| −0.06536 | Post NgtdmTextures.Complexity |

US, ultrasound; LNM, lymph node metastasis; LASSO, least absolute shrinkage and selection operator.

**Table S2.** Radiomic features for predicting lateral LNM and weighting coefficients after LASSO regression

| Lasso weighted coefficient | US radiomics feature in formula |
| --- | --- |
| 0.01168 | M spiculation-1 |
| 0.18408 | M spiculation-2 |
| 0.08111 | M overlap Area |
| -0.10231 | Mean of Non-Similarity of Tumor |
| 0.08141 | Cal SD of Roundness |
| 0.01375 | Cal Circumference of All Calcification |
| -0.08020 | Cal Area c min |
| 0.01659 | Cal Area c max |
| -0.01439 | SD of Posterior Area Contrast |
| -0.02654 | SD of Non-Similarity of Posterior Area |
| 0.14546 | Histogram t MAD |
| -0.12041 | Elliptic-Normalized Angle |
| 0.07381 | Glszm.Gray-Level Nonuniformity |
| 0.20612 | Glszm.Low-Gray-Level Zone Emphasis |
| -0.00963 | Glszm.Zone-Size Variance |
| -0.02101 | Posterior Histogram t Entropy |

US, ultrasound; LNM, lymph node metastasis; LASSO, least absolute shrinkage and selection operator.
